# Anti-*Toxoplasma gondii* and Anti-*Neospora caninum* Antibodies in Urban Traction Equids in Northeast Brazil: Seroprevalence and Risk Factors

**DOI:** 10.3390/tropicalmed8040234

**Published:** 2023-04-20

**Authors:** Paulo Wbiratan Lopes Costa, Clarisse Silva Menezes Oliveira, Roberto Alves Bezerra, Felipe Boniedj Ventura Alvares, Victor Hugo Alves Sousa Formiga, Marianne Rachel Domiciano Dantas Martins, Thais Ferreira Feitosa, Vinícius Longo Ribeiro Vilela

**Affiliations:** 1 Pos-Graduating Program in Science and Animal Health, Universidade Federal de Campina Grande, Patos 58708-110, Paraíba, Brazil; paulo.lopes@unipe.edu.br (P.W.L.C.); marianne.martins@unipe.edu.br (M.R.D.D.M.); 2 Department of Veterinary Medicine, Instituto Federal de Educação, Ciência e Tecnologia da Paraíba, Sousa 58814-000, Paraíba, Brazil; clarissesmenezeso@gmail.com (C.S.M.O.); felprathalos@gmail.com (F.B.V.A.); victorallves.96@gmail.com (V.H.A.S.F.); thais.feitosa@ifpb.edu.br (T.F.F.)

**Keywords:** donkeys, horses, mules, neosporosis, toxoplasmosis

## Abstract

The aim of this study was to describe the prevalence of anti-*T. gondii* and anti-*N. caninum* antibodies in equids that carry out traction work in Northeastern Brazil, and to establish the potential risk factors associated with seropositivity for these agents. Blood samples were collected from 322 traction equids (horses, donkeys and mules) in urban areas of 16 municipalities in the State of Paraíba, Brazil. The samples were sent for serological diagnosis using the Immunofluorescence Antibody Test (IFAT). Epidemiological questionnaires were given to the owners to assess the possible risk factors associated with infections. It was observed that 13.7% (44/322, CI: 10.9–16.5) of the equids tested positive for anti-*T. gondii* antibodies and 5% (16/322, CI: 2.6–7.4) tested positive for anti-*N. caninum* antibodies. Conducting traction work for over four years was considered a risk factor associated with *T. gondii* infection (odds ratio: 6.050; CI: 4.38–8.54, *p* = 0.025). There were no risk factors associated with *N. caninum* infection. It was concluded that traction equids have a significant prevalence of anti-*T. gondii* and anti-*N. caninum* antibodies in urban areas in the State of Paraíba, with an identified risk factor for seropositivity for anti-*T. gondii* as conducting traction work for more than four years.

## 1. Introduction

Traction equids have a relevant social and economic role for vulnerable families, especially in Northeast Brazil, as these animals carry out long journeys daily [1]. Among the several diseases that can affect equids, we highlight *Toxoplasma gondii* and *Neospora caninum* infections, which are caused by obligate intracellular protozoa parasites. As a result of these diseases, equids can be affected as intermediate hosts and suffer reproductive and neurological disorders, such as miscarriage, neonatal mortality and encephalomyelitis [2,3,4].

In Brazil, equids are highly exposed to infections by *T. gondii* and *N. caninum*, being important in the epidemiology of these parasites’ infections [5]. Epidemiological studies to detect anti-*T. gondii* antibodies in rural horses showed frequencies ranging from 10.3% to 11.6% in the states of Pará and Paraíba, respectively [6,7]. For infections by *N. caninum*, the positivity frequencies in rural horses ranged from 5.6% to 23.9% in the States of Pará and Minas Gerais, respectively [7,8]. However, there is a scarcity of studies evaluating *T. gondii* and *N. caninum* infections in donkeys and mules, or evaluating the epidemiological profile of these infections in traction equids raised in urban environments.

The aim of this study was to evaluate the prevalence of anti-*T. gondii* and anti-*N. caninum* antibodies and to find potential risk factors associated with seropositivity for these agents in urban traction equids in Northeast Brazil.

## 2. Materials and Methods

### 2.1. Study Area and Sampling

**Figure 1:**
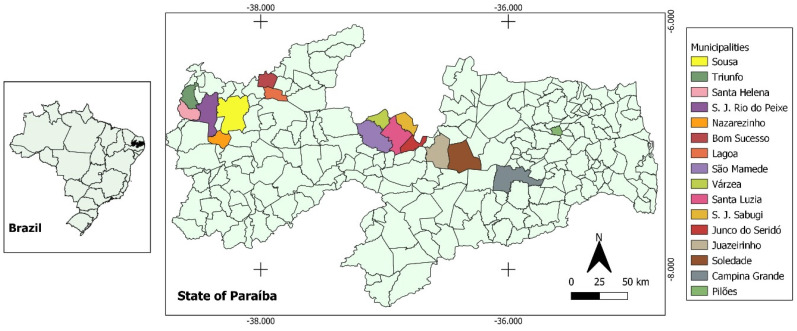
Geographic location of municipalities in the state of Paraíba, Northeast Brazil, where samples of urban traction equids were collected to assess the prevalence and risk factors associated with positivity for anti-*T. gondii* and anti-*N. caninum* antibodies.

Serum samples from traction equids were collected in 16 municipalities in the semi-arid region of the state of Paraíba, Brazil (Figure 1).

To determine the minimum number of animals to be sampled, simple random sampling was used:

$$n=\frac{Z^{2}\times P\left(1-P\right)}{d^{2}}$$

*n* = sampling number; *Z* = normal distribution value for the 95% confidence level; *P* = expected prevalence of 10.3%—*T. gondii*; 5.6%—*N. caninum* [7]; *d* = 5% sampling error.

To perform adjustments for finite populations, the following formula was applied:

$$najus=\frac{N\times n}{N+n}$$

*najus* = adjusted sample size; *N* = total population size; *n* = initial sample size.

The adjustment of the population sample size considered the total population of equids in the state of Paraíba, which was taken to be 98,584 animals (IBGE; available at https://cidades.ibge.gov.br/brasil/pb/pesquisa/24/27745, accessed on 5 March 2023). Thus, the minimum number of animals required for participation in the study was 142 for anti-*T. gondii* prevalence and 81 for anti-*N. caninum* prevalence. However, 322 samples were collected.

### 2.2. Sample Selection

**Figure 2:**
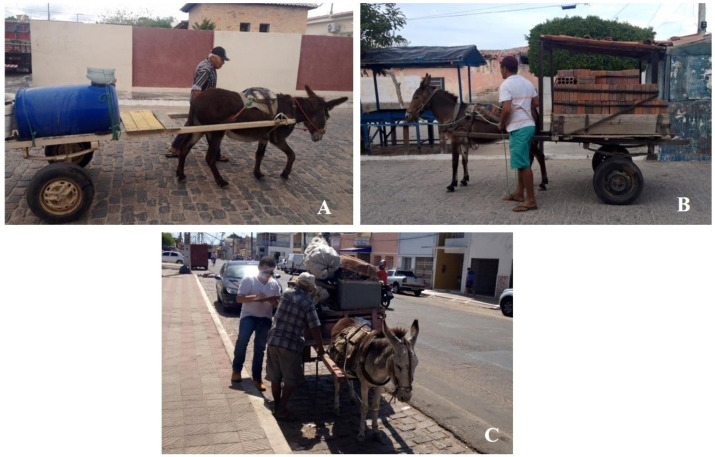
Owners were approached for consent, questionnaire application and blood collection of traction equids during urban work in the state of Paraíba, Northeastern Brazil. (**A**): Traction donkey transporting a water tank for human and animal consumption; (**B**): Traction mule transporting bricks for civil construction; (**C**): Traction donkey transporting recyclable material.

Convenience sampling was used, meaning that the equids included in the research were performing traction work in urban areas during active searches in the municipalities. The minimum age of the horses used in the study was one year old, regardless of race or sex (Figure 2).

After taking assigned consent from the owner of the animal, external jugular venipuncture was used to collect 5 mL of blood from each animal. The samples were individually identified, packaged and sent to the Laboratory of Immunology and Infectious Diseases (LIID) at the Veterinary Hospital at the Instituto Federal da Paraíba (IFPB), Sousa campus, where they were centrifuged at 2500 rpm for 10 min, and then the sera were stored in 2 mL microtubes and frozen at −20 °C [6].

### 2.3. Serological Analyses

The Immunofluorescence Antibody Test (IFAT) was used to detect anti-*T. gondii* antibodies based on the work of Langoni et al. [8]. *T. gondii* taquizoytes (RH strain) were used as antigens. Positive and negative control sera from horses were included in each slide. Sera were considered positive when *T. gondii* tachyzoites showed total peripheral fluorescence at a 1:64 cut-off [9].

To detect anti-*N. caninum* antibodies, the IFAT was based on the work of Conrad et al. [10]. *N. caninum* taquizoytes (NC-1 strain) were used as antigens. Positive and negative control sera from horses were included in each slide. Sera were considered positive when *N. caninum* tachyzoites showed total peripheral fluorescence at a 1:50 cut-off [11].

The conjugate (anti-horse IgG, labelled with fluorescein isothiocyanate, Sigma^®^, St. Louis, MO, USA) was used at a 1:1500 dilution in pH 7.2 phosphate-buffered solution (PBS) containing 0.01% Evans blue. Positive samples were titrated from sequential dilutions on the basis of two until negative.

### 2.4. Epidemiological Questionnaire

Pre-structured questionnaires were given to owners to assess the risk factors associated with *T. gondii* and *N. caninum* infections. The variables included for analysis were related to species (donkey, mule, horse), breed, sex, age and time of traction activity. Questions related to environmental management, contact with other species (dogs, cats, sheep, cattle, pigs, wild animals and other equids), nutritional management, type of food and storage, as well as health and reproductive information were asked.

### 2.5. Statistical Analysis

Descriptive statistical analysis was used to calculate the frequencies of the results obtained in the serological test. Risk factors associated with *T. gondii* and *N. caninum* infections were assessed using data from epidemiological questionnaires in two stages: univariate and multivariate analysis. In the univariate analysis, each independent variable was cross-correlated with the dependent variable (seropositivity), and those with a *p*-value ≤ 0.2, according to the chi-square test or Fisher’s exact test [12], were selected for multivariate analysis using multiple logistic regression [13]. In order to verify some collinearity between the data, a correlation test was applied whereby if the correlation coefficient was greater than 0.9, one of the variables was eliminated using the criteria of biological plausibility. To verify the fit of the model, the chi-square parameters and the omnibus test were used. The significance level adopted in the multiple analyses was 5%. The results were analysed using GraphPad Prism version 8.0.0 for Windows, GraphPad Software, San Diego, CA, USA.

## 3. Results

It was observed that, among the 322 traction equids evaluated, 13.7% (44/322, CI: 10.9–16.5) tested positive for anti-*T. gondii* antibodies, and for anti-*N. caninum* antibodies the prevalence was 5% (16/322, CI: 2.6–7.4). The seropositivity by species for both anti-*T. gondii* and anti-*N. caninum* antibodies is shown in Table 1. There were no animals simultaneously positive for anti-*T. gondii* and anti-*N. caninum* antibodies.

The anti-*T. gondii* antibody titrations ranged from 1:64 to 1:1024 and from 1:50 to 1:100 for the anti-*N. caninum* antibody titrations (Table 2).

In the univariate analysis for the anti-*T. gondii* antibodies’ positivity, only the variables of age and time of work were selected for multiple logistic regression (*p* ≤ 0.20) (Table 3). In the multivariate analysis, a time of work variable higher than four years was considered a risk factor associated with anti-*T. gondii* positivity (odds ratio: 6.05).

In the univariate analysis for the anti-*N. caninum* antibodies positivity, only the variable sex was selected for multiple logistic regression (*p* ≤ 0.20) (Table 4). However, there were no risk factors associated with anti-*N. caninum* antibodies.

## 4. Discussion

The prevalence rates of 13.7% for anti-*T. gondii* and 5% for anti-*N. caninum* antibodies were similarly observed in the northern region of Brazil, with a prevalence of 10.3% (134/1298) for anti-*T. gondii* and 5.6% (73/1298) for anti-*N. caninum* [7]. Lower seroprevalence in horses was observed in the State of São Paulo, Southeast Brazil, with prevalence values of 0.9% (1/116) for anti-*T. gondii* and 2.6% (3/116) for anti-*N. caninum* [14]. On the other hand, in studies conducted in the Northeast and Midwest regions of Brazil, the prevalences were 28.5% (129/453) for anti-*T. gondii* in horses and 2% (7/333) for anti-*N. caninum* in donkeys [15,16]. The intensity of *T. gondii* and *N. caninum* infections varies between different locations, exposures and study focuses. Surveyed population, management conditions and environmental characteristics may contribute to the variation in seropositivity between studies [17,18].

This study provides relevant information about the seroprevalence of anti-*T. gondii* (12%) and anti-*N. caninum* (5.1%) in mules, since, among the equids, it is the least studied [7,19,20]. However, mules, despite having a predisposition to infertility [21], are able to travel long distances in urban and rural areas [22]. As animals that use force to perform traction work, exposure to these agents may decrease their production and productivity.

The results of these anti-*T. gondii* and anti-*N. caninum* antibody studies demonstrated that most seropositive animals had low titers, mainly 1:64 (70.4%) for anti-*T. gondii* and 1:50 (93.7%) for anti-*N. caninum*. Low antibody titers were also observed by James et al. [23], in which 73% (239/328) of the animals titrated below 1:100 for anti-*T. gondii*, and by Bartová et al. [24], who observed that all positive animals for anti-*T. gondii* (3%; 19/643) and for anti-*N. caninum* (2.3%; 15/6430) antibodies titrated up to 1:100. Low titers for anti-*T. gondii* in horses should not be ignored [25], as they commonly develop lower antibody titers [26,27]. In infections by other pathogens, they found that high titers of antibodies were observed after recent contact with the antigen, indicating acute infection, in addition to low titers in chronic infections, denoting old contact with the agent [28,29].

A time of work of more than four years was considered a risk factor associated with *T. gondii* infection in traction equids (Odds ratio: 2.89). Li et al. [30] observed that the older horses become, the more they are exposed to jobs that involve travelling long distances, and therefore are predisposed to have contact with food and water contaminated by *T. gondii* oocystis. In our study, the results may have occurred because traction equids are commonly exposed to a wide variety of urban environments, making long journeys, carrying a high load weight, maintaining contact with other species of animals, and are associated with precarious care provided by owners, all of which increase the possibility of being infected by *T. gondii* and/or *N. caninum.*

There were no risk factors associated with positive results for anti-*N. caninum* antibodies in the studied animals. However, it was observed that the majority of positive equids maintained contact with dogs (87.5%). It is possible that oocysts can be excreted by dogs in food, water and pasture, becoming sources for *N. caninum* infections [31,32].

The expressive prevalence of anti-*T. gondii* antibodies in equids denotes that adequate prevention and control measures must be taken. Although it does not occur frequently, the literature mentions that there may be human infections from the ingestion of *T. gondii*-infected equine meat [25,33], and that older animals are most commonly used for food [24]. Even though the consumption of equid meat is not common in Brazil, there is an emphasis on the export of this product [17]. The prevalence found in the present study for infections by *N. caninum* emphasises the need for prevention, since it can cause reproductive neosporosis [34,35].

## 5. Conclusions

It was concluded that traction equids had a significant prevalence of anti-*T. gondii* and anti-*N. caninum* antibodies in urban areas in the semi-arid region of Northeast Brazil. Traction work for more than four years was a risk factor associated with seropositivity for anti-*T. gondii*. Surveillance and epidemiological control measures, such as registration and periodic serological testing, should be taken in order to establish what actions are required to reduce infections by these protozoa.

## Figures and Tables

**Table 1:** Prevalence of anti-*Toxoplasma gondii* and anti-*Neospora caninum* antibodies in urban traction horses, donkeys and mules in the semi-arid region of Northeast Brazil.

Variable/ Category	Total Equids	Positives Anti-*T. gondii*	% (CI)	*p*	Positives Anti-*N. caninum*	% (CI)	*p*
Species							
Horse	76	12	15.8 (9.7–21.9)	0.748	3	4 (2.2–5.8)	0.889
Donkey	91	13	14.3 (10.8–17.8)		5	5.5 (3.6–7.4)	
Mule	155	19	12.2 (10–14.4)		8	5.1 (3.5–6.7)	

CI: Confidence interval at 95% probability.

**Table 2:** Distribution of anti-*Toxoplasma gondii* and anti-*N. caninum* antibody titrations by Immunofluorescence Antibody Test (IFAT) in traction equids in the semi-arid region of Northeastern Brazil.

**Positivity of Anti-*T. gondii* Antibodies**					
Titration	1:64	1:128	1:256	1:512	1:1.024
Total (%)	31 (70.4)	9 (20.4)	1 (2.3)	1 (2.3)	2 (4.6)
**Positivity of Anti-*N. caninum* Antibodies**					
Titration	1:50	1:100	1:200	1:400	1:800
Total (%)	15 (93.7)	1 (6.3)	-	-	-

**Table 3:** Univariate and multivariate analyses of risk factors associated with the positivity for anti-*Toxoplasma gondii* antibodies in traction equids in the semi-arid region of Northeastern Brazil.

Variable/Category	Univariate Analysis			Multivariate Analysis		
	Total of Equids	Positive Anti-*T. gondii* (%)	*p*	OR	CI	*p*
Sex						
Male	138	19 (13.8)	>0.999			
Female	184	25 (13.6)				
Age						
≤4 years	72	14 (19.4)	0.177 *	0.460	0.20–1.05	0.063
5–9 years	130	13 (10)		Ref.		
10–13 years	120	17 (14.2)		0.841	0.37–1.88	0.671
Feed						
Pasture	144	20 (13.9)	0.992			
Pasture + Corn	140	19 (13.6)				
Pasture + Commercial Food	38	5 (13.1)				
Contact with Cats						
Yes	274	39 (14.2)	0.649			
No	48	5 (10.4)				
Time of Work						
≤3 years	40	1 (2.5)	0.025 *	Ref.		
≥4 years	282	43 (15.2)		6.050	4.38–8.54	<0.0001

OR: odds ratio; CI: confidence interval; Ref.: reference value. * Variables that presented *p* values ≤ 0.20 according to the chi-square test and/or Fisher’s exact test.

**Table 4:** Univariate and multivariate analyses of risk factors associated with the positivity for anti-*Neospora caninum* antibodies in traction equids in the semi-arid region of Northeastern Brazil.

Variable/Category	Univariate Analysis			Multivariate Analysis		
	Total of Equids	Positives Anti-*N. caninum* (%)	*p*	OR	CI	*p*
Sex						
Male	138	4 (2.9)	0.195 *	1.139	0.48–2.63	0.7595
Female	184	12 (6.5)		Ref.		
Age						
≤4 years	72	2 (2.8)	0.475			
5–13 years	129	6 (4.6)				
10–13 years	120	8 (6.7)				
Feed						
Pasture	144	8 (5.5)	0.882			
Pasture + Corn	140	6 (4.2)				
Pasture + Commercial Food	38	2 (5.3)				
Contact with dogs						
Yes	237	14 (5.9)	0.254			
No	85	2 (2.3)				
Time of Work						
≤3 years	40	1 (2.5)	0.703			
≥4 years	282	15 (5.3)				

OR: odds ratio; CI: confidence interval; Ref.: reference value. * Variables that presented *p* values ≤ 0.20 according to the chi-square test and/or Fisher’s exact test.

## Data Availability

Not applicable.
